# Understanding the Solvent Molecules Induced Spontaneous Growth of Uncapped Tellurium Nanoparticles

**DOI:** 10.1038/srep32631

**Published:** 2016-09-07

**Authors:** Jun Liu, Changhao Liang, Xiaoguang Zhu, Yue Lin, Hao Zhang, Shouliang Wu

**Affiliations:** 1 Key Laboratory of Materials Physics, Institute of Solid State Physics, Chinese Academy of Sciences, Hefei, 230031, China; 2 Hefei National Laboratory for Physical Sciences at the Microscale, University of Science and Technology of China, Hefei, 230026, China

## Abstract

Understanding the thermodynamic behavior and growth kinetics of colloidal nanoparticles (NPs) is essential to synthesize materials with desirable structures and properties. In this paper, we present specific uncapped Te colloidal NPs obtained through laser ablation of Te in various protic or aprotic solvents. At ambient temperature and pressure, the uncapped Te NPs spontaneously exhibited analogous evolution and growth of “nanoparticle-nanochain-agglomerate-microsphere” in different solvents. The distinctive growth kinetics of the formation of nanochains strongly depended on the polarity and dielectric constant of solvent molecules. The growth rate of agglomerates and microspheres was closely related to the zeta potential of the colloidal solution of Te nanochains and the average size of Te agglomerates. Furthermore, the resulting uncapped Te NPs and Te nanochains displayed a prominent size-dependent and structure-inherited chemical reductive ability. These findings provide insights into the growth of active uncapped nanoparticles in various dispersion media. This study also provides an alternative route in designing novel nanostructures of alloys, telluride, and functional composites using Te as a unique reactive precursor.

Investigating the growth behavior of nanoparticles (NPs) in colloidal solution and exploring their intrinsic physicochemical properties have become interesting points in nanomaterial and nanotechnology studies^1^,^2^,^3^,^4^,^5^. By comparison with bulk materials, NPs exhibit many unique properties derived from the excellent size and/or surface effects of NPs^6^,^7^. In most “bottom-up” chemical syntheses (as shown in Fig. 1), owing to rapid nucleation and growth, capping surfactant ligands or polymers on NP surfaces are usually introduced to control particle size and shape, and prohibit agglomeration^8^,^9^,^10^. As these agents are passivated on the surfaces of NPs, the surface free energy is reduced, which thermodynamically stabilizes the NPs with defined size and shape in colloidal solution. However, these agent-capped NPs may conceal some intrinsic growth characteristics or fundamental physicochemical properties that depend on the high surface free energy. If the NPs in the colloidal solution are not capped with any surfactant ligands, NPs with high active surfaces may experience completely different growth behaviors and exhibit unexpected physicochemical properties in the colloidal solution^11^,^12^.

In our previous work, we obtained pure Ge NPs using laser ablation in liquid (LAL)^13^. Under ambient temperature and pressure, uncapped Ge NPs in water showed spontaneous structural evolution. In this process, the initial amorphous structure gradually evolved into a metastable tetragonal structure and eventually transformed into a stable cubic structure. The phase transition of such Ge NPs can be stopped if the Ge NPs are kept as dry powders. These findings indicated that uncapped NPs have high surface activity, and the interactions between the highly active surface and surrounding water molecules may be the driving force for the phase transformation. Zhang *et al.* synthesized uncapped ZnS NPs in methanol and found that the initial ZnS NPs distributed in a disordered structure in methanol can transform into a more crystalline sphalerite-like structure in response to the addition of water at room temperature^14^. The phase transformation of ZnS NPs was ascribed to the stronger interactions between water and ZnS than that between methanol and ZnS. Interestingly, Leekumjorn *et al.* found that changes in surrounding molecules can only affect the hydrodynamic size, colloidal stability, and sedimentation aggregation behavior of NPs in surfactant ligand-capped NPs^15^. That is because there are only interactions between solvent molecules and capping ligands, the NPs are protected by capping ligands, the size or structures are not readily influenced.

Obviously, the interactions between uncapped NPs and solvent molecules are more complex than those between surface-capped NPs and solvent molecules. The surrounding molecules play important roles in the structural evolution, phase transformation, or intrinsic properties of uncapped NPs. The capping ligands on the NP surfaces prevent the study of the spontaneous growth process and restrict the exploration of the intrinsic physicochemical properties derived from highly active surfaces. Moreover, normal wet chemical synthesis is not suitable for preparing uncapped NPs in colloidal solutions. Thus, finding an appropriate route to obtain NPs with uncapped surfaces, exploring the interactions between uncapped NPs and solvent molecules, and investigating the thermodynamic behavior of uncapped NPs in various circumstances are needed to produce materials with specific structures and unique properties dependent on surface effects.

With above motivations, in this study, using element Te as a typical example, we use a “top-down” laser ablation in liquids (LAL) method to obtain uncapped tellurium (Te) NPs, particularly in five kinds of solvents including protic or aprotic solvents, such as water, methanol, ethanol, acetone and dichloromethane. Here the initial Te colloidal solutions was obtained by laser ablation of Te target under the same laser parameters and same volume of solvents, expectedly, the initial colloidal solutions show almost identical concentrations of Te NPs, which was evaluated by Inductively Coupled Plasma Mass Spectrometer (ICP) measurements (Supplementary Table S1). However, in subsequent ageing processes, these solvents display interesting effects on the nucleation and growth of Te nanostructures. Correspondingly, the intrinsic physicochemical properties of uncapped Te with highly active surfaces were explored.

## Results

### Structure evolution of uncapped Te NPs in various solvents

The schematic for the formation of uncapped Te NPs and the succeeding structural evolution in solvents are illustrated in Fig. 2. LAL is a fast kinetic process. As the focused pulse laser attacks the target, the excited plasma plume with high pressure, high temperature, and high species density is generated at the solid–liquid (target solvents) interface. This process occurs in a very short life-time within a 100 ns scale. When the plasma breaks down, the active Te species stripped from the Te target may rapidly cool, nucleate, grow and form NPs dispersed in the surrounding solvents^16^,^17^. By prolonging the duration of laser ablation, the concentration of uncapped Te NPs increase, the size and structure of uncapped Te NPs should be analogous during every laser ablation pulse. In our experiments, a black plume was injected into the solvent through laser ablation of Te target in liquids for 2 s, which demonstrated the formation of Te NPs or clusters. We then obtained samples from the plume and immediately performed transmission electron microscopy (TEM) analysis (Fig. 2A1–E1, Supplementary Fig. S1A–E). The corresponding HRTEM images (Fig. 3A1–E5) indicated that the uncapped Te NPs obtained in CH_3_OH, CH_3_CH_2_OH, CH_3_COCH_3_, and CH_2_Cl_2_ assumed an amorphous structure, whereas the uncapped Te NPs in H_2_O were well crystallized. The lattice plane was indexed with (012) facets and interplanar spacing of 2.36 Å, which implied that the uncapped Te NPs formed in water exhibited higher surface energy and faster crystallization than others. On the basis of the calculation and fitting of over 200 NPs from the TEM images, the uncapped Te NPs obtained average sizes of 1.79, 1.78, 2.31, 2.00, and 2.00 nm in H_2_O, CH_3_OH, CH_3_CH_2_OH, CH_3_COCH_3_, and CH_2_Cl_2_, respectively (Supplementary Fig. S2A_1_–E_1_ and Table S2).

The Te NPs obtained in various solvents exhibited remarkable activity because of the active surfaces. Under ambient temperature and pressure, they spontaneously underwent analogous “nanoparticle-nanochain-agglomerate-microsphere” structural evolution.

**Figure 1:**
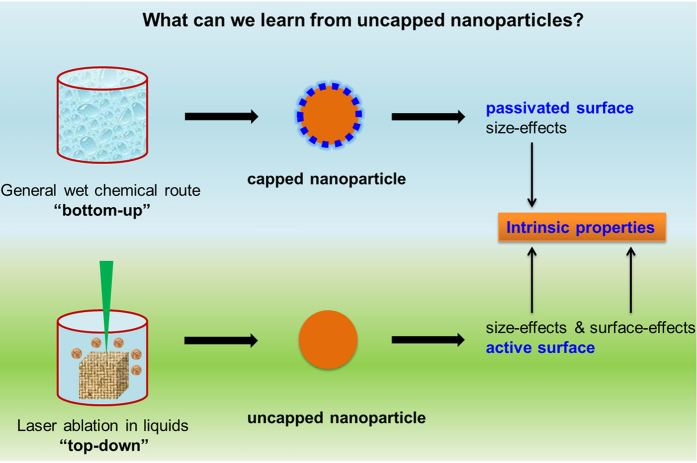
Difference between intrinsic properties of capped and uncapped NPs in colloidal solution.

**Figure 2:**
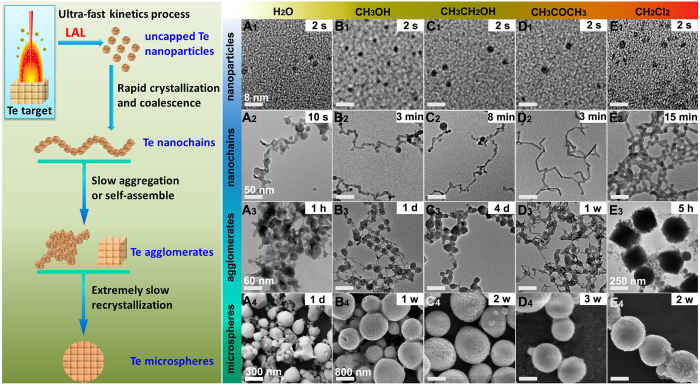
Schematic of spontaneous growth of uncapped Te NPs in various solvents and corresponding data on Te microstructures. TEM images of uncapped Te NPs (**A**_**1**_–**E**_**1**_), nanochains (**A**_**2**_–**E**_**2**_), and agglomerates (**A**_**3**_–**E**_**3**_). SEM images of microspheres (**A**_**4**_–**E**_**4**_) in five kinds of solvents, namely, H_2_O, CH_3_OH, CH_3_CH_2_OH, CH_3_COCH_3_, and CH_2_Cl_2_. Scale bar: 8 nm for **A**_**1**_–**E**_**1**_, 50 nm for **A**_**2**_–**E**_**2**_, 60 nm for **A**_**3**_–**D**_**3**_, 250 nm for **E**_**3**_, 300 nm for A_4_, and 800 nm for **B**_**4**_–**E**_**4**_. Time of taking sample to perform TEM characterization: 2 s for A_1_–E_1_; 10 s for A_2_, 3 min for **B**_**2**_ and **D**_**2**_, 8 min for **C**_**2**_, 15 min for E_2_; 1 h for A_3_, 1 day for **B**_**3**,_ 4 days for **C**_**3**_, 1 week for **D**_**3**_, 5 h for E_3_; 1 day for **A**_**4**_, 1 week for **B**_**4**_, 2 weeks for **C**_**4**_ and **E**_**4**_, and 3 weeks for **D**_**4**_.

First, the uncapped Te NPs crystallized and self-assembled into Te nanochains. According to the TEM images displayed in Fig. 2A2–C2, the Te nanochains that were formed in H_2_O, CH_3_OH, and CH_3_CH_2_OH were shaped in irregular curves, not in straight lines. The nanochains were composed of many crystallized Te NPs with average sizes of 12.97, 8.24, and 6.67 nm in H_2_O, CH_3_OH, and CH_3_CH_2_OH, respectively (Supplementary Fig. S2A_2_–C_2_ and Table S2). By contrast, the Te nanochains in CH_2_Cl_2_ with a diameter of 9.08 nm (Supplementary Fig. S2E_2_ and Table S2) connected together and formed a net-like morphology (Fig. 2E2). In particular, the Te nanochain formed in CH_3_COCH_3_ comprised several straight lines that were connected with each other from different angles (Fig. 1D2). However, the average diameter of these Te nanochains was only 4.48 nm, which was much smaller than that of others (Supplementary Fig. S2D_2_ and Table S2). X-ray diffraction (XRD) patterns (Supplementary Fig. S3) showed that the Te nanochains formed in various solvents were well crystallized, and the characteristic peaks could be indexed to the hexagonal structure of Te (JCPDS File No. 36-1452)^18^. However, two extra diffraction peaks located at 2θ degree of 26.18° and 29.91° in every XRD pattern belonged to the TeO_2_ index at the (110) and (102) lattice planes (JCPDS File No. 65-8053), respectively. These peaks were attributed to the partial surface oxidation of Te nanochains caused by the small amounts of dissolved oxygen in the solvents.

**Figure 3:**
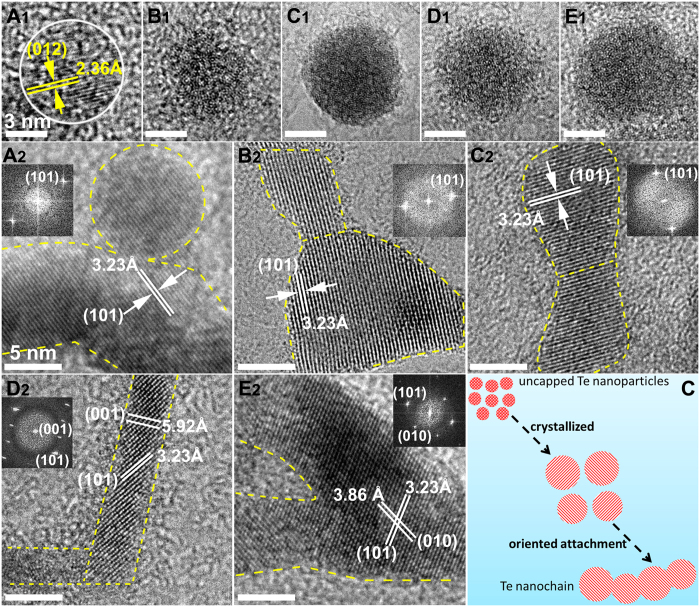
HRTEM images of an individual uncapped Te NP (**A**_**1**_–**E**_**1**_) and sectional Te nanochain (**A**_**2**_–**E**_**2**_) in H_2_O, CH_3_OH, CH_3_CH_2_OH, CH_3_COCH_3_, and CH_2_Cl_2_. Scale bar: 3 nm for **A**_**1**_–**E**_**1**_; 5 nm for **A**_**2**_–**E**_**2**_. Formation of Te nanochains dominated by the typical oriented attachment (OA) growth mechanism (**F**).

HRTEM images of Te nanochains in H_2_O (Fig. 3A2) showed that the uncapped Te NPs coalesced with each other and displayed an oriented attachment (OA) in the (101) plane with a lattice spacing of 0.323 nm. We clearly observed a common crystallographic orientation at the boundary among the NPs. This growth process precisely coincided with the typical OA growth mechanism (Fig. 3F)^19^,^20^. Interestingly, the same OA growth process occurred for the uncapped Te NPs in the four other kinds of solvents (Fig. 3B2–E2). In general, OA growth is a thermodynamically metastable state between the nucleation stage and Ostwald ripening (OR) growth stage in a colloidal system. At the initial stage, relatively small NPs with strongly charged surface surfactants hinder OR growth to exclusively promote OA growth^20^,^21^. However, our experiments showed that the uncapped Te NPs still spontaneously attached to each other to form nanochains in an oriented direction despite the absence of capping surfactants or ligands. We supposed that the ultra-small size distribution of Te NPs induced a high surface free energy that accelerated the coalescence of adjacent NPs. Moreover, the uncapped surface of Te NPs caused the numerous unsaturated coordination sites on the surface to easily absorb or bond with solvent molecules. This strong interaction between the surface of uncapped Te NPs and solvent molecules should be one of the key factors for the generation of Te nanochains via the OA-based growth mechanism.

**Figure 4:**
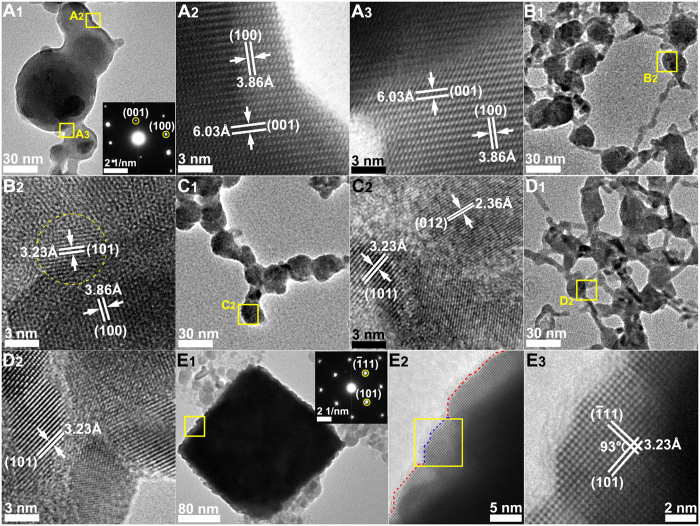
TEM images, HRTEM images, and related SAED patterns of Te agglomerates formed in various solvents. H_2_O (**A**_**1**_–**A**_**3**_), CH_3_OH (**B**_**1**_,**B**_**2**_), CH_3_CH_2_OH (**C**_**1**_,**C**_**2**_), CH_3_COCH_3_ (**D**_**1**_,**D**_**2**_) and CH_2_Cl_2_ (**E**_**1**_–**E**_**3**_).

Subsequently, the Te nanochains that formed in different solvents gradually aggregated and grew into agglomerates with different morphologies (Fig. 2A3–E3). In H_2_O, the Te agglomerates were large spheres with small tails (Fig. 4A1) and an average diameter of 34.16 nm (Supplementary Fig. S2A_3_ and Table S2). The small tail attached to the large sphere was actually a short Te nanochain, which confirmed the formation of Te agglomerates in water. The corresponding SAED pattern (inset in Fig. 4A1) proved that the single crystalline structure of the Te agglomerates was formed in H_2_O as the electron beam was focused along the [010] zone axis. The HRTEM images (Fig. 4A2,A3) of the attachment area between the neighboring particles of the agglomerate marked with a yellow box in Fig. 4A1 indicated that the connections were both single crystalline structures. Moreover, the two main facets were indexed to (001) facets with interplanar spacing of 6.03 Å and (100) facets with interplanar spacing of 3.86 Å, which agreed with the SAED analysis results. By contrast, all Te agglomerates generated in CH_3_OH, CH_3_CH_2_OH, and CH_3_COCH_3_ (Fig. 3B1–D1) appeared like a ball of strings intertwined by Te nanochains to construct a sphere-like structure with average diameters of 20.53, 20.29, and 17.31 nm, respectively (Supplementary Fig. S2B_3_–D_3_ and Table S2). Several short Te nanochains connected the sphere-like structures. Related HRTEM images (Fig. 4B2–D2) marked by yellow boxes in Fig. 4B1–D1 showed that these Te agglomerates with a typical polycrystalline structure were generated by the aggregation of many Te NPs.

Surprisingly, the Te nanochains in CH_2_Cl_2_ formed regular Te nanocubes (Fig. 2 E_3_, Supplementary Fig. S4A) with an average size of 320 nm (Supplementary Fig. S2E_3_ and Table S2). An individual Te nanocube exhibited a rough surface made of compactly assembled small Te nanocrystals, as shown in a highly magnified SEM image (Supplementary Fig. S4B). A TEM image of a single Te nanocube (Fig. 4E1) also revealed uneven edges and a solid inner structure. Corresponding SAED patterns (inset in Fig. 4E1) along the [−1−21] zone axis demonstrated the single crystalline nature of Te nanocubes. The HRTEM image (Fig. 4E2) of the area marked by a yellow box in Fig. 4 E1 showed the side of a Te nanocube orderly integrated by several Te NPs, which were free from defects and dislocations. The magnified HRTEM image (Fig. 4E3) of the area marked by a yellow box in Fig. 4E2 showed two lattice planes ([101] and [−111]) with the same interplanar spacing of 3.25 Å and an intersection angle of 93°, which precisely coincided with the SAED pattern. The above microstructure analyses demonstrated that all large Te agglomerates gradually grew at the expense of small NPs on Te nanochains, regardless of solvent type; although final products displayed variety in morphology and crystalline structure, all these structural evolutions could be classified under the OR crystal growth mechanism^22^,^23^.

Finally, all Te agglomerates sequentially grew into large Te microspheres (Fig. 2A4–E4; Supplementary Fig. S2A_4_–E_4_ and Table S2) with prolonged aging time. The oxidation of the Te nanostructures should not be neglected during the long growth process^24^,^25^. Compared with the Te nanochains in H_2_O, the corresponding XRD patterns of Te microspheres (Supplementary Fig. S5) showed that the intensity of diffraction peaks at the (101) lattice plane decreased, whereas those at the (110) and (102) lattice planes in TeO_2_ sharply increased. However, the XRD patterns of the Te microspheres generated in the four other organic solvents showed no evident increase in the intensity of the diffraction peaks of the TeO_2_ phase. These results implied that the surface of Te nanostructures prepared in H_2_O exhibited higher activity than that prepared in other solvents, which resulted in the oxidation of more Te nanostructures in water to TeO_2_. These TeO_2_ products did not only result from the oxidization of the available dissolved oxygen, but they may have also originated from an oxidation reaction between the Te nanostructures and water molecules, as shown in equation (1):


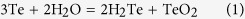


### Distinct growth kinetics of uncapped Te NPs in different solvents

**Figure 5:**
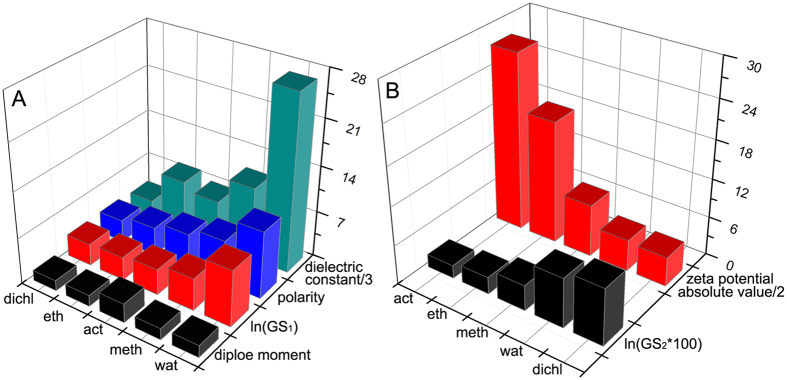
Investigation of spontaneous growth rate for uncapped Te nanoparticles and nanochains in various solvents. (**A**) Correlation between ln(GS_1_) and properties of solvent molecules, such as polarity, dielectric constant, and dipole moment. (**B**) Correlation between ln(GS_2_*100) and absolute zeta potential. “wat,” “meth,” “eth,” “act,” and “dichl” are abbreviations for water, methanol, ethanol, acetone, and dichloromethane, respectively.

Although the uncapped Te NPs in various solvents underwent similar growth behavior, a notable difference in growth kinetics was observed. For instance, Fig. 2A2–E2 show that the uncapped Te NPs in H_2_O were integrated into nanochains within 10 s; however, the growth rates in CH_3_OH, CH_3_CH_2_OH, CH_3_COCH_3_, and CH_2_Cl_2_ were relatively slow and required several minutes to generate nanochains. In addition, the formation of Te nanospheres in H_2_O or the generation of Te nanocubes in CH_2_Cl_2_ simply required 1 or 5 h, whereas the formation of Te agglomerates in the other solvents needed a couple of days to one week. Evidently, the solvents played a critical role in the growth kinetics of Te nanostructures. To determine how the solvent molecules affect the growth kinetics of uncapped Te NPs, the formation of Te nanochains by the OA mechanism was defined as Stage 1, and the generation of Te agglomerates and microspheres by the OR mechanism was defined as Stages 2 and 3, respectively. The corresponding growth rates of the different stages (GS) were roughly calculated by ∆average size/∆time (nm/hour) and termed as GS_1_, GS_2_, and GS_3_ (Supplementary Table S3). As shown in Supplementary Table S3, the growth rate of Te nanochains formation (GS_1_) was determined using the natural logarithm. The related column graph (Fig. 5A) shows that ln(GS_1_) declined in the following order: H_2_O > CH_3_OH > CH_3_COCH_3_ > CH_3_CH_2_OH > CH_2_Cl_2_.

Obviously, the distinction in growth rate of Te nanochains was strongly affected by the type of solvent molecules. In order to deeply understand the relationship between growth rate of Te nanochains and solvent molecules, we analyzed possible interactions between uncapped Te NPs and solvent molecules in detail. In colloidal solution, Derjaguin-Landau-Verwey-Overbeek (DLVO) theory was usually used to describe the stabilization of nanoparticles in solution^26^,^27^. The DVLO theory assumes that the interaction potential energy (EDLVO) of two spherical nanoparticles is the sum of the attractive van der Waals energy (*E*_*a*_) of attraction between adjacent particles and the repulsive electrostatic energy (*E*_*r*_) of the electric double layers, which is given in equation (2):


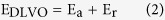


However, the dynamic size change of Te nanostructures with non-spherical shapes cannot be investigated by the DLVO model, which is appreciate for spherical nanoparticles^20^. But the van der Waals attractive energy and repulsive electrostatic energy should be considered in the growth kinetics of Te nanochains. It means that van der Waals (vdW) interaction and Coulombic interaction (CI) between different assembly objects should be mainly considered^20^,^28^,^29^. The vdW interaction between two spherical particles is given by equation (3)^30^:


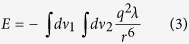


where *ν*_*1*_ and *ν*_*2*_ are the respective volumes of the two particles, *r* is the distance between the two particles, *q* is the atomic concentration of the particles, and *λ* is the vdW constant. For the generation of Te nanochains, the concentration and size distribution of uncapped Te NPs (Supplementary Fig. S2A_1_–E_1_) was approximate to each other in various solvents. It imply that the contribution of vdW to ln(GS_1_) could be considered identical in each solvent. Therefore, CI should directly determine the distinction in growth rate of Te nanochains in different solvents, which was described by Coulomb’s law in equation (4)^31^:


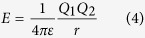


where *ε* is the dielectric constant of the medium, *Q*_*1*_ and *Q*_*2*_ are the charge values of two particles, *r* is the separation between two particles. CI induces repulsive force which would hinder coalescence and aggregation of Te NPs and decrease the growth rate of Te nanochains. Therefore, according to equation (4), ln(GS_1_) is directly proportional to the charge value of Te NPs and inversely proportional to the dielectric constant of solvent molecules. By comparison (as shown in Fig. 5A), it is found that the ln(GS_1_) is almost varied inversely to the dielectric constant of solvent molecules^32^ (Supplementary Table S4) except the one in CH_3_COCH_3_, which exhibited a faster growth rate but smaller dielectric constant than those in CH_3_CH_2_OH. Therefore, we suppose that the charge value of Te NPs formed in CH_3_COCH_3_ might be larger than that in CH_3_CH_2_OH. However, as the short lifespan of Te NPs in solvents, it is hard to get the charge value of Te NPs. Moreover, there is no surfactant or electrolyte added in solvents, the charge value of Te NPs is thus derived from the ionization of solvent molecules, which is closely correlated with intrinsic properties of solvent molecules, such as polarity. Interesting, the polarity of CH_3_COCH_3_ is just larger than that in CH_3_CH_2_OH^32^ (Supplementary Table S4). Importantly, the variation tendency of ln(GS_1_) well coincided with the change in polarity of solvent molecules (Fig. 5A). Thus we believe that the distinctive growth kinetics during the formation of nanochains was strongly dependent on the polarity and dielectric constant of solvent molecules.

The formation of Te agglomerates from nanochains followed the OR growth mechanism, and its corresponding growth rate (GS_2_) differed under various solvents. The aggregation and sedimentation of Te colloidal solution also occurred during this growth process. For convenient comparisons, the value of GS_2_ was multiplied by 100 and the natural logarithm was obtained (Table S3). The values of ln(GS_2_ × 100) were in the following order: CH_2_Cl_2_ > H_2_O > CH_3_OH > CH_3_CH_2_OH > CH_3_COCH_3_, as shown in Fig. 5B. It is well know that zeta (ζ) potential is a key factor in the stability of colloidal solution. Electric double layers surrounding the colloidal NPs hinder their aggregation or sedimentation in solvents. As a result, the high absolute value of the zeta potential (critical value of 30 mV) causes a strong repulsive force that stabilizes the colloidal NPs^13^,^33^. The Te nanochains generated in various solvents presented zeta (ζ) potential values of −9.7, −16.2, −37.9, −54.8, and 9.1 mV for H_2_O, CH_3_OH, CH_3_CH_2_OH, CH_3_COCH_3_, and CH_2_Cl_2_, respectively (Supplementary Fig. S6). According to the data shown in Fig. 5B, the variation tendency of the OR growth rate of Te agglomerates in various solvents was inversely proportional to the absolute value of the zeta potential. Thus, a high zeta potential resulted in stable Te nanochains in solvents, as well as slow growth kinetics in the formation of Te agglomerates. High zeta potential of the Te nanochains in CH_3_COCH_3_ provided good stability for 1 week with minimal agglomeration, whereas the Te nanochains formed in H_2_O or CH_2_Cl_2_ underwent rapid aggregation and growth because of the low zeta potential.

The formation of microspheres was also dominated by the OR growth mechanism. As time progressed during the coarsening of agglomerates, the interaction between the solvent molecules and the surface of Te nanostructures became weak. Desorption of solvent molecules occurred on the surface of the Te nanostructures, but the growth rate of microspheres (GS_3_) was dependent on the interactions between solvent molecules and Te nanostructures. Supplementary Table S3 displays the natural logarithm value of growth rate for Te microspheres [ln(GS_3_)] arranged in the order of H_2_O > CH_3_OH > CH_3_CH_2_OH > CH_3_COCH_3_ > CH_2_Cl_2_. The lowest growth rate of Te microspheres was found in CH2Cl2 because the large Te nanocubes exhibited the lowest surface free energy as a growth driving force.

## Discussion

**Figure 6:**
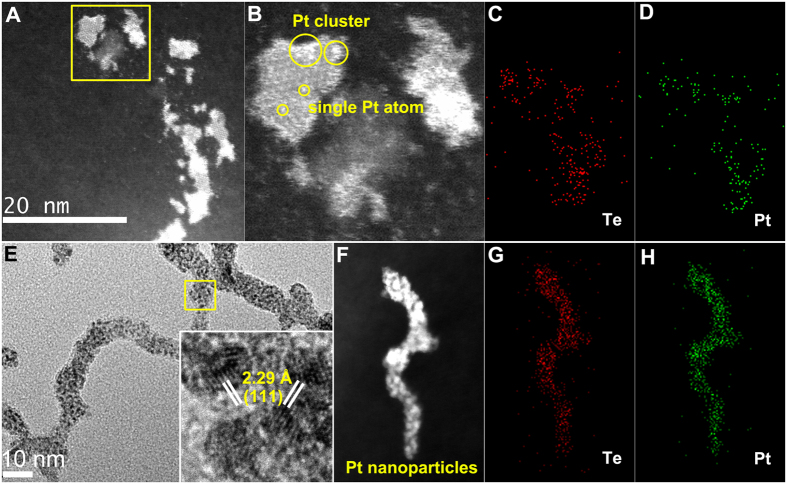
Structure and component analysis of Pt-located Te NPs and Pt-Te hybrids nanochains. Pt-located Te NPs: HADDF images (**A**,**B**), elemental mapping image (**C**,**D**) of Te and Pt. Pt-Te hybrid nanochains: (**E**) TEM image, inset is HRTEM image of the yellow box marked area; (**F**) HADDF image; (**G**,**H**) elemental mapping image of Te and Pt.

To elucidate the size and surface-dependent chemical reactivity between Te nanomaterials and H_2_PtCl_6_ aqueous solution, we chose uncapped Te NPs formed in water and Te nanochains prepared in acetone as reduced precursors to react with H_2_PtCl_6_ aqueous solution at room temperature. After the reaction, we obtained two distinct Pt-Te hybrid structures. The uncapped Te NPs displayed ultra-high chemical reductive ability and reacted *in situ* with PtCl_6_^2−^ to generate Pt-located Te NPs during laser ablation for 5 s (Fig. 6A). High-resolution HADDF (Fig. 6B) showed very small sizes of these Pt NPs, some of which were Pt clusters or single Pt atoms. The corresponding elemental analysis (Fig. 6C,D) indicated that Pt atoms were well dispersed on Te NPs, and the atomic ratio of Pt and Te was 1:6 for Pt-Te hybrid NPs (Supplementary Fig. S7A). The Te nanochains obtained in our experiments not only exhibited higher reactive ability but also displayed a structure-inherited reductive ability as sacrificed templates. By using Te nanochains generated in acetone as precursors, Pt-Te hybrid structures with chain-like morphologies were obtained, as shown in Fig. 6E. Many Pt NPs with an average size of 2 nm were formed on the surface of Te nanochains. The inset of the HRTEM image shows a lattice plane with interplanar spacing of 2.29 Å that indexed with the Pt (111) lattice plane. Elemental mapping analysis further demonstrated that the Pt NPs generated on Te nanochains were well dispersed. Energy dispersive X-ray spectroscopy (EDX) analysis (Supplementary Fig. S7B) indicated that the atomic ratio of Pt and Te was 1:1 for Pt-Te hybrids.

In the past decade, Yu *et al.* synthesized numerous 1D Te nanostructures, such as nanoribbons, nanotubes, and nanowires^34^,^35^,^36^. Based on the thermodynamically unstable nature of ultra-fine Te nanowires, they prepared series-based materials, such as noble metals, alloys, telluride, and carbon-based hybrids^37^,^38^,^39^,^40^. For instance, to obtain Pt nanowires^37^ or PtPdTe alloys^38^, the ultra-fine Te nanowires were used as templates mixed with Pt or Pd precursors, the mixture solution was shaken by an Innova 40 Benchtop Incubator Shaker for at 50 °C for 8 or 13 hours. By comparison, the Pt-located Te NPs or Pt-Te hybrid nanochains in our experiments were obtained by reacting with H2PtCl6 at room temperature and pressure without any extra driving forces. The uncapped Te NPs or Te nanowires obtained in this study exhibited small size distributions and high Gibbs free energy, which induced unstable surfaces and high chemical reactivity. By adjusting the concentration of metal ions, reaction time, or Te nanostructures, we could readily synthesize Te-based hybrid nanomaterials with the desired morphology and components.

In summary, we prepared uncapped Te NPs in five kinds of solvents by LAL. The uncapped Te NPs spontaneously underwent similar “nanoparticle-nanochain-agglomerate-microsphere” growth behavior in different solvents at room temperature. However, the growth kinetics for the structural evolution of uncapped Te NPs in various solvents was distinctive. The OA growth rate of Te nanochains strongly depended on the polarity and dielectric constant of solvent molecule. The growth rate of the generation of agglomerates and microspheres was dominated by the zeta potential of the Te nanochain colloidal solution and the average size of agglomerates. These findings provide important insights into the influence of surrounding molecules in the growth process of uncapped NPs in colloidal solutions. In addition, by using the obtained uncapped Te NPs and nanochains as reaction templates, Pt-Te hybrids with different sizes and morphologies could be obtained at room temperatures. These results supply an alternative route for designing novel nanostructures of alloys, telluride, and functional composites.

## Methods

### Preparation of uncapped Te NPs by LAL

A Te target with hexagonal phase (99.99%) was immersed in 15 mL of solvent and ablated using a 1064 nm Nd:YAG pulse laser with a pulse duration of 10 ns and pulse energy of 30 mJ to obtain uncapped Te NPs. Under various solvents, namely, H_2_O, CH_3_OH, CH_3_CH_2_OH, CH_3_COCH_3_, and CH_2_Cl_2_, uncapped Te NPs in different dispersion media were obtained.

### Investigation of structural evolution for uncapped Te NPs in solvents

All experiments were performed at ambient temperature and pressure. To investigate the structural evolution of uncapped Te NPs, we obtained samples from solvents at different times (Table 1) and observed the microstructure under TEM.

Uncapped Te NPs: After laser ablation, Te was targeted for 2 s in every solvent. The sample was obtained immediately with a pipette and dropped on a copper mesh, which was used for structural characterization.

Te nanochains: The laser ablation time was 10 s in H_2_O and 3 min in CH_3_OH and CH_3_COCH_3_. The sample was obtained immediately and dropped on a copper mesh. With laser ablation duration of 3 min for CH_3_CH_2_OH and CH_2_Cl_2_, the obtained colloidal solution was stored in a dark chamber without heating or stirring for 5 and 12 min, respectively. The sample was then dropped on a copper mesh.

Te agglomerates and microspheres: With laser ablation duration of 3 min in every solvent, the obtained colloidal solutions of Te nanochains were stored in a dark chamber without heating or stirring. In certain time intervals, we investigated the size and structural evolution of uncapped Te NPs and found an approximate critical time for Te agglomerates and microspheres in different solvents, as shown in Table 1.

### Chemical reductive ability of uncapped Te NPs and nanochains

Uncapped Te NPs reacted with H_2_PtCl_6_ aqueous solution: The Te target was placed in 15 mL of 10^−6^ g/mL H_2_PtCl_6_ aqueous solution and ablated for 10 s using a 1064 nm Nd:YAG pulse laser with a pulse duration of 10 ns and pulse energy of 30 mJ. An *in-situ* reaction occurred between the produced uncapped Te NPs and PtCl_6_^2−^ in colloidal solution. After laser ablation for 10 s, the samples were immediately obtained and dropped on a copper mesh, which was used for structural and component characterization.

Te nanochains reacted with H_2_PtCl_6_ aqueous solution: First, the Te target was placed in acetone and ablated for 3 min using a 1064 nm Nd:YAG pulse laser with a pulse duration of 10 ns and pulse energy of 30 mJ. Subsequently, 5 mL of Te nanochains colloidal solution was used as reaction precursor and mixed with 5 mL of 2 × 10^−4^ moL/L H_2_PtCl_6_ aqueous solution. The mixture was intensely stirred for 12 h at room temperature and ambient pressure without heating treatment. Finally, we obtained the black-color flocculent products.

### Characterization of structure and morphology

A Philips X’Pert system with Cu Kα radiation (λ = 1.5419 Å, scanning rate 1.0°/min) was used to perform XRD on the prepared Te samples. Field emission SEM (Sirion 200 FEG) was used to observe the morphologies of Te products. A TEM system (JEOL, JEM-2010) with 200 kV acceleration voltage was used to obtain the regular structural information of the products. Another TEM system (JEM-ARM 200F) was used to visualize the nanostructures of Pt-Te hybrids. EDX elemental mapping images were used to determine the distribution of elemental Te or Pt in Pt-Te hybrids. MALVERN instrument (Zetasizer 3000 HSa) was used to measure the zeta (f) potential of Te colloidal solution.

## Additional Information

**How to cite this article**: Liu, J. *et al.* Understanding the Solvent Molecules Induced Spontaneous Growth of Uncapped Tellurium Nanoparticles. *Sci. Rep.* **6**, 32631; doi: 10.1038/srep32631 (2016).

## Supplementary Material

Supplementary Information

## Figures and Tables

**Table 1:** Time nodes for uncapped Te NPs, Te nanochains, agglomerates, and microspheres were obtained from different solvents.

	H_2_O	CH_3_OH	CH_3_CH_2_OH	CH_3_COCH_3_	CH_2_Cl_2_
Nanoparticles	2 seconds	2 seconds	2 seconds	2 seconds	2 seconds
Nanochains	10 seconds	3 minutes	8 minutes	3 minutes	15 minutes
Agglomerates	1 hour	1 day	4 days	1 week	5 hours
Microspheres	1 day	1 week	2 weeks	3 weeks	2 weeks
