# Supplementary material for: Understanding the Solvent Molecules Induced Spontaneous Growth of Uncapped Tellurium Nanoparticles
Source: Sci Rep. 2016 Sep 7;6:32631. doi: 10.1038/srep32631 (PMC5013520; doi:10.1038/srep32631)
Supplement: Supplementary Information [file srep32631-s1.pdf]

# Supplementary Information

## Understanding the Solvent Molecules Induced Spontaneous Growth of Uncapped Tellurium Nanoparticles

Jun Liu[1], Changhao Liang*[1,2], Xiaoguang Zhu[1], Yue Lin[2], Hao Zhang[2] and Shouliang Wu[1]

[1]Key Laboratory of Materials Physics, Institute of Solid State Physics, Chinese Academy of Sciences, Hefei, 230031, China

[2]Hefei National Laboratory for Physical Sciences at the Microscale, University of Science and Technology of China, Hefei, 230026, China
Correspondence and requests for materials should be address to C. H. L (chliang@issp.ac.cn)

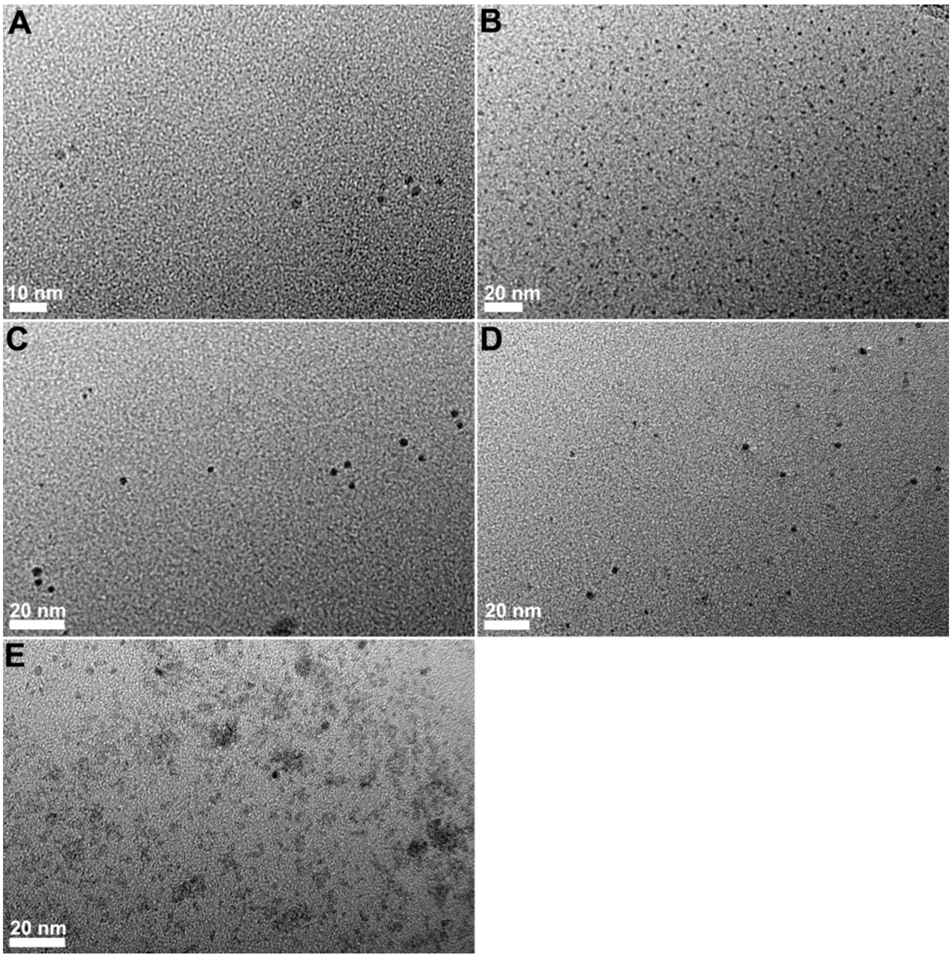


**Figure S1 TEM images of uncapped Te nanoparticles generated in $H_2O$ (A), $CH_3OH$ (B), $CH_3CH_2OH$ (C), $CH_3COCH_3$ (D) and $CH_2Cl_2$ (E), respectively**

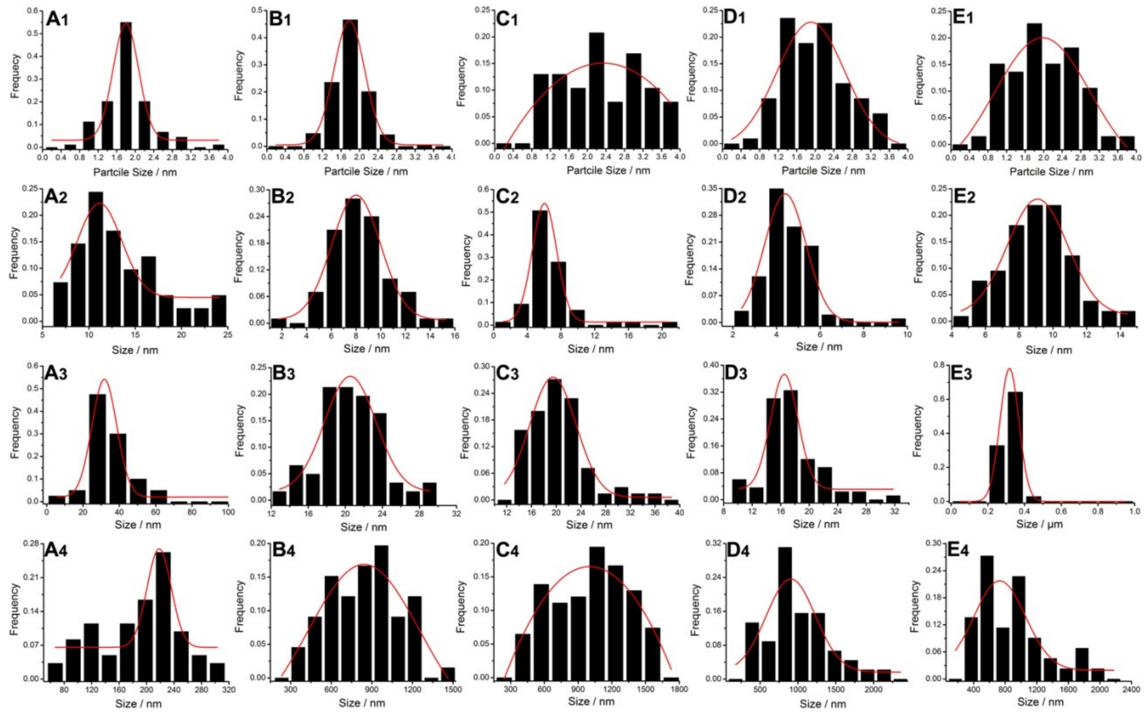


**Figure S2 Size distribution histograms of uncapped Te nanoparticles ($A_1$-$E_1$), nanochains ($A_2$-$E_2$), agglomerates ($A_3$-$E_3$) and microspheres ($A_4$-$E_4$) generated in $H_2O$, $CH_3OH$, $CH_3CH_2OH$, $CH_3COCH_3$ and $CH_2Cl_2$, respectively.**

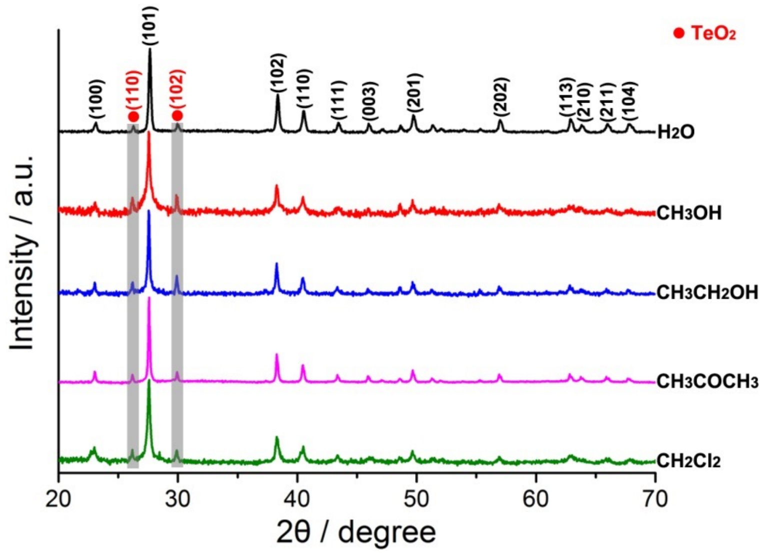


**Figure S3 XRD patterns of Te nanochains formed in $H_2O$, $CH_3OH$, $CH_3CH_2OH$, $CH_3COCH_3$ and $CH_2Cl_2$. Red dots marked peaks indexed to $TeO_2$.**

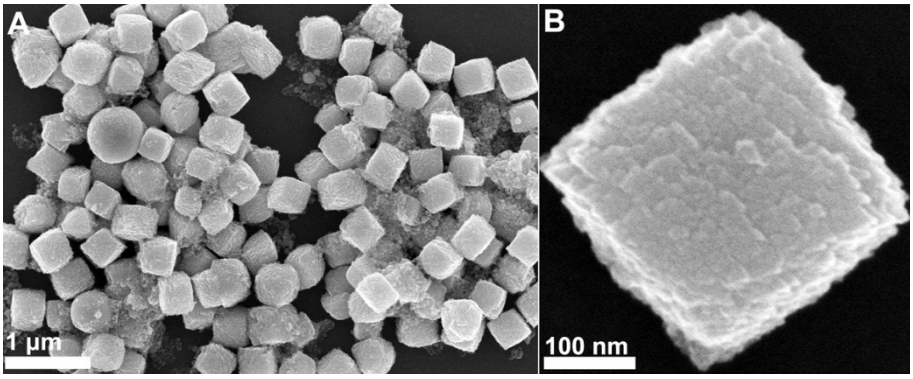


**Figure S4 Low- and high magnification SEM images of Te nanocubes formed in $CH_2Cl_2$.**

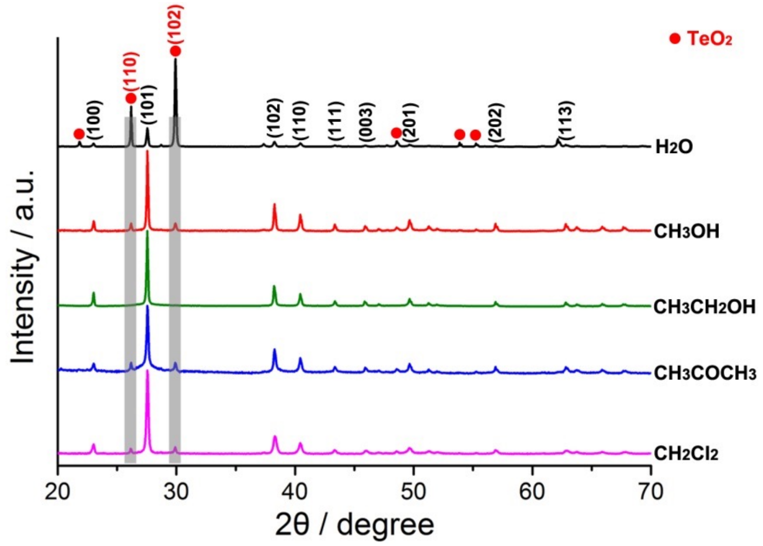


**Figure S5 XRD patterns of Te microspheres generated in $H_2O$, $CH_3OH$, $CH_3CH_2OH$, $CH_3COCH_3$ and $CH_2Cl_2$. Red dots marked peaks indexed to $TeO_2$.**

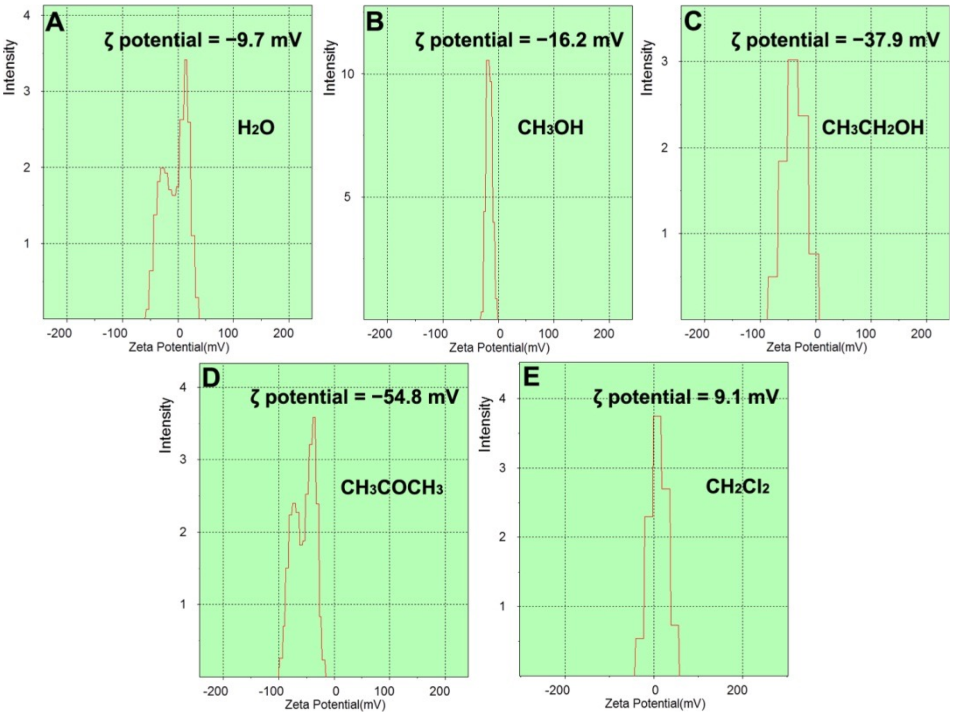


**Figure S6 Zeta potential of Te nanochains colloidal solution in various solvents: $H_2O$ (A), $CH_3OH$ (B), $CH_3CH_2OH$ (C), $CH_3COCH_3$ (D) and $CH_2Cl_2$ (E) in sequence.**

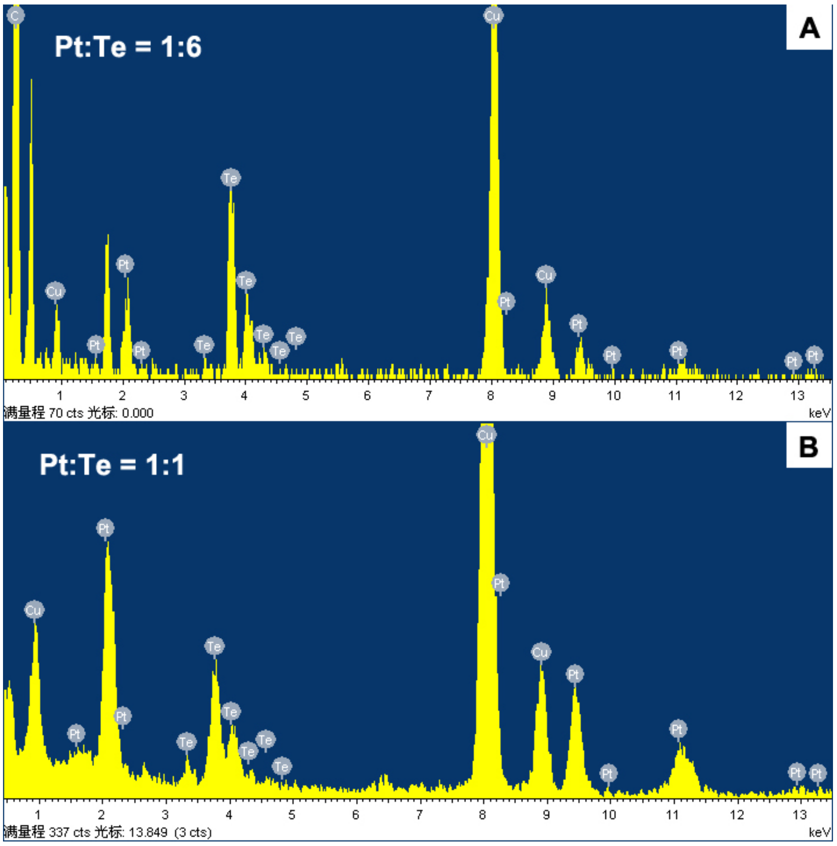


**Figure S7 EDX spectra of Pt located Te nanoparticles and Pt-Te hybrid structures.**

**Table S1*** Concentration of initial Te nanoparticles colloidal solution in five kinds of solvents, which was performed by Inductively Coupled Plasma Mass Spectrometer (ICP).

| Solvents | Concentration of Te nanoparticles colloidal solution (mg/L) |
|---|---|

| Solvents | 1 | 2 | 3 | Average value | Standard Deviation |
|---|---|---|---|---|---|
| $H_2O$ | 24.11 | 24.11 | 24.22 | 24.15 | 0.052 |
| $CH_3OH$ | 24.93 | 25.00 | 25.22 | 25.05 | 0.123 |
| $CH_3CH_2OH$ | 26.00 | 25.14 | 25.91 | 25.68 | 0.386 |
| $CH_3COCH_3$ | 25.67 | 25.69 | 25.81 | 25.72 | 0.062 |
| $CH_2Cl_2$ | 24.20 | 24.90 | 24.71 | 24.60 | 0.296 |

*Note: Laser ablation in liquids is a special method for preparing colloidal solution, the concentration of colloidal solution is strongly correlated with laser parameters, including wavelength of pulse laser, pulse laser energy (or fluence), ablation time, volume of liquids. In our experiment, the Te target was immersed in 15 mL of solvents and ablated using a 1064 nm Nd: YAG pulse laser with a pulse duration of 10 ns and pulse energy of 30 mJ to obtain Te nanoparticles. Under room temperature and pressure, the Te nanoparticles in various solvents obtained with same ablation time show the same initial concentrations.

**Table S2 *Average size and standard deviation (σ) of uncapped Te nanoparticles, nanochains, agglomerates and microspheres generated in $H_2O$, $CH_3OH$, $CH_3CH_2OH$, $CH_3COCH_3$ and $CH_2Cl_2$.**

| solvents | nanoparticles | | nanchains | | agglomerates | | microspheres | |
|---|---|---|---|---|---|---|---|---|
| | average size | σ | average size | σ | average size | σ | average size | σ |
| $H_2O$ | 1.79 | 0.50 | 12.97 | 4.34 | 34.16 | 11.19 | 190.07 | 58.51 |
| $CH_3OH$ | 1.78 | 0.38 | 8.24 | 2.23 | 20.53 | 3.33 | 840.14 | 254.83 |
| $CH_3CH_2OH$ | 2.31 | 0.86 | 6.67 | 2.81 | 20.29 | 4.49 | 1006.35 | 332.94 |
| $CH_3COCH_3$ | 2.00 | 0.66 | 4.48 | 1.05 | 17.31 | 3.82 | 990.37 | 409.06 |
| $CH_2Cl_2$ | 2.00 | 0.70 | 9.08 | 1.98 | 320.00 | 51.69 | 885.60 | 419.89 |

*Note: The average size and standard deviation of different Te nanostructures was collected on the basis of the calculation and fitting of over hundreds of Te nanostructures from dozens of TEM or SEM images. The unit of average size and standard deviation are both nanometers (nm).

**Table S3 Growth rate of Te nanchains, agglomerated and microspheres in various solvents.** Formation of Te nanochains by OA mechanism was designed as Stage 1, generation of Te agglomerates and microspheres was designed as Stage 2 and Stage 3, respectively. The corresponding growth rate of different Stage (GS) was calculated by Δaverage size/ Δtime (nm/hour) and defined as $GS_1$, $GS_2$ and $GS_3$, respectively. To make convenient comparison, the value of $GS_1$ and $GS_3$ was both took the natural logarithm. For drawing related column bar graph, the value of $GS_2$ multiplies by 100 and then took the natural logarithm.

| solvents | $\ln(GS_1)$ | $\ln(GS_2)$ | $\ln(GS_2 \times 100)$ | $\ln(GS_3)$ |
|---|---|---|---|---|
| $H_2O$ | 8.52 | 3.06 | 7.66 | 1.91 |
| $CH_3OH$ | 4.87 | −0.67 | 3.94 | 1.74 |
| $CH_3CH_2OH$ | 3.49 | −1.95 | 2.65 | 1.41 |
| $CH_3COCH_3$ | 3.92 | −2.57 | 2.03 | 1.06 |
| $CH_2Cl_2$ | 3.35 | 4.16 | 8.77 | 0.54 |

**Table S4 Fundamental physicochemical properties of different solvents, including $H_2O$, $CH_3OH$, $CH_3CH_2OH$, $CH_3COCH_3$ and $CH_2Cl_2$.**

| solvents | polarity | dielectric constant | dipole moment (Debye) | viscosity (cp 20°C) |
|---|---|---|---|---|
| $H_2O$ | 10.2 | 80 | 1.85 | 1.0 |
| $CH_3OH$ | 6.6 | 33 | 1.7 | 0.6 |
| $CH_3CH_2OH$ | 4.3 | 24.3 | 1.69 | 1.2 |
| $CH_3COCH_3$ | 5.4 | 20.7 | 2.88 | 0.32 |
| $CH_2Cl_2$ | 3.4 | 9.08 | 1.6 | 0.44 |
